# Supplementary material for: Global freshwater fish invasion linked to the presence of closely related species
Source: Nat Commun. 2024 Feb 15;15:1411. doi: 10.1038/s41467-024-45736-8 (PMC10869807; doi:10.1038/s41467-024-45736-8)
Supplement: Supplementary file 1 — Supplementary Information [file 41467_2024_45736_MOESM1_ESM.pdf]

Supplementary Information

Global freshwater fish invasion linked to the presence of closely related species

Meng Xu[1,2,3*], Shao-peng Li[4], Chunlong Liu[5], Pablo A. Tedesco[6], Jaimie T. A. Dick[7], Miao Fang[1,2,3], Hui Wei[1,2,3], Fandong Yu[1,2,3], Lu Shu[1,2,3], Xuejie Wang[1,2,3], Dangen Gu[1,2,3*], Xidong Mu[1,2,3*]

[1]Pearl River Fisheries Research Institute, Chinese Academy of Fishery Sciences

[2]Key Laboratory of Prevention and Control for Aquatic Invasive Alien Species, Ministry of Agriculture and Rural Affairs, Guangzhou, China

[3]Key Laboratory of Alien Species and Ecological Security (CAFS), Chinese Academy of Fishery Sciences, Guangzhou, China

[4]Zhejiang Tiantong Forest Ecosystem National Observation and Research Station, School of Ecological and Environmental Sciences, East China Normal University, Shanghai, China

[5]The Key Laboratory of Mariculture, Ministry of Education, College of Fisheries, Ocean University of China

[6]UMR EDB, IRD 253, CNRS 5174, UPS, Université Toulouse 3 Paul Sabatier, Toulouse, France

[7]Institute for Global Food Security, School of Biological Sciences, Queen's University Belfast, Belfast, UK

* Corresponding author | Meng Xu, Email: xumeng@prfri.ac.cn; Dangen Gu, Email: gudangen@163.com; Xidong Mu, Email: muxd@prfri.ac.cn

This PDF file includes:

Fig. S1 to S8

Table S1 to S3

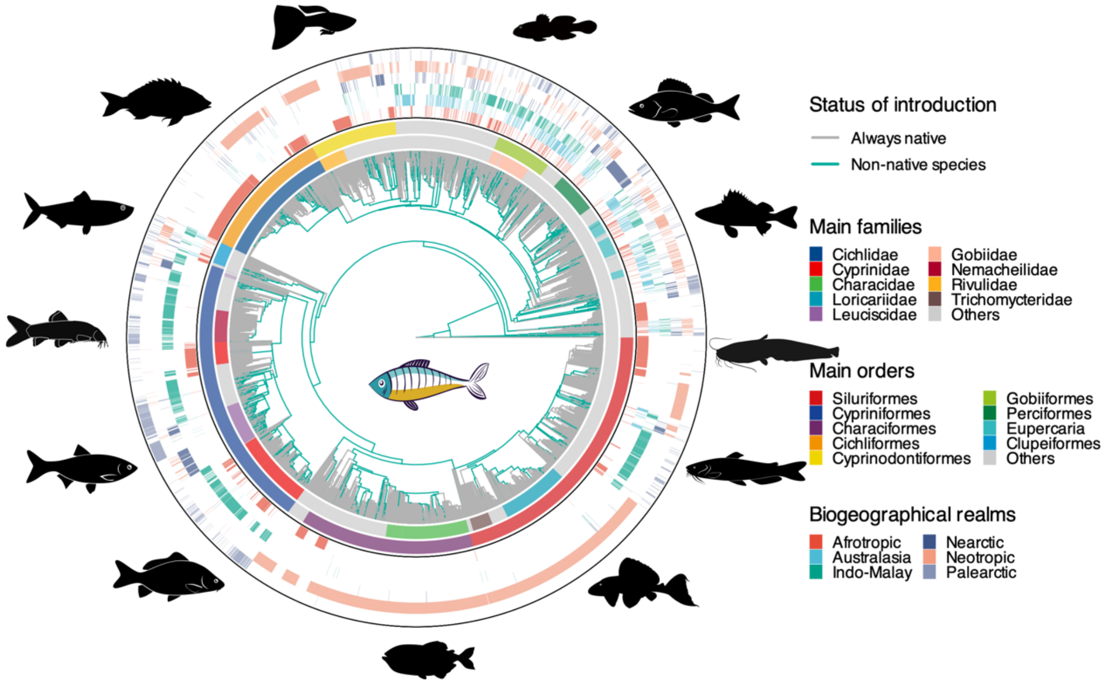

Fig. S1 | Phylogenetic tree for 14708 freshwater fish species occurring in 3008 river basins worldwide. This tree illustrates the evolutionary relationships among freshwater fish species worldwide, of which 597 species (green branches) have been introduced into other countries or translocated among river basins within a country. The first inner ring represents the main families that the fish species belong to, the second inner ring denotes the main orders, and the outermost ring indicates the major biogeographical realms of the world.

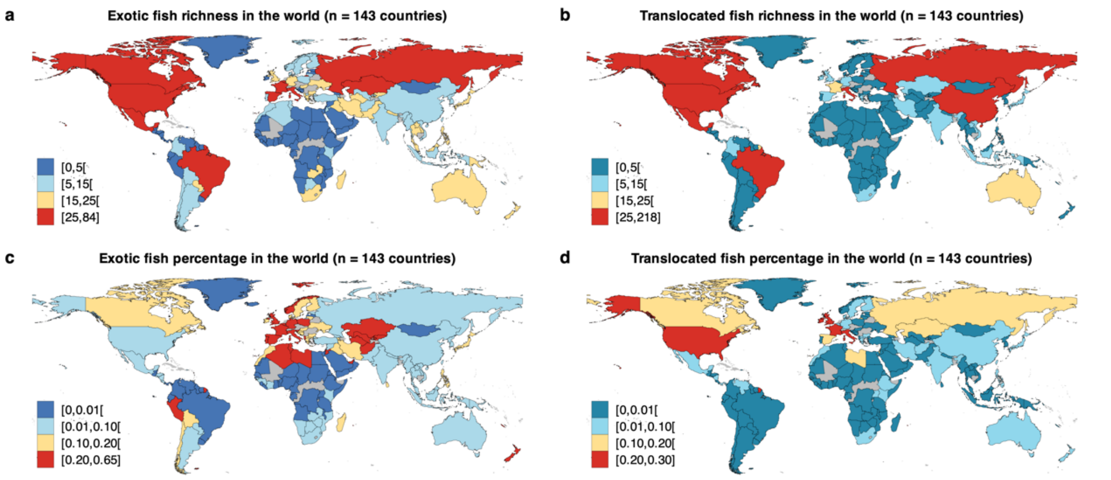

Fig. S2 | Geographical distribution of exotic and translocated freshwater fish species across different countries. **a** Geographical pattern of exotic fish richness. **b** Geographical pattern of translocated fish richness. **c** Geographical pattern of exotic fish percentage. **d** Geographical pattern of translocated fish percentage. The percentage is referred to the ratio of non-native species richness to the total species richness in each country. The number of countries used for assessing these patterns is shown at the top of each panel.

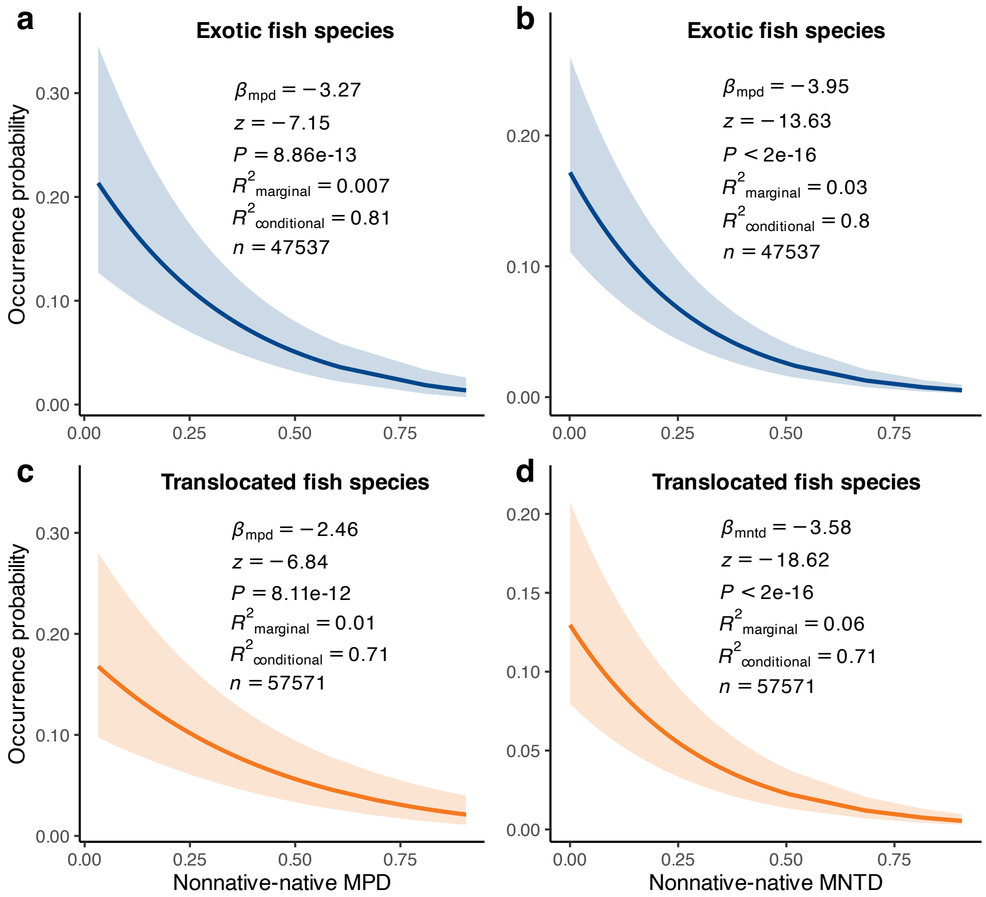

Fig. S3 | Relationships between the probability of non-native fish occurrence and nonnative-native phylogenetic distance after excluding the non-native species within the two dominant families, Cyprinidae and Salmonidae. a The variation in the occurrence probability of exotic fish species with the mean phylogenetic distance (MPD) between exotic and native fish species. **b** The variation in the occurrence probability of exotic fish species with the nearest phylogenetic distance (MNTD) between exotic and native fish species. **c** The variation in the occurrence probability of translocated fish species with the MPD between translocated and native fish species. **d** The variation in the occurrence probability of translocated fish species with the MNTD between translocated and native fish species. Statistical tests and predictive curves (with 95% confidence intervals) were obtained using generalized linear mixed models (GLMMs), while assuming a binomial error distribution. Statistical significance (*P* values), variance explained ($R^2_{marginal}$ for the fixed effect and $R^2_{conditional}$ for both the fixed and random effects), and sample size (*n*) are presented in the figure. Blue and orange colors are used to highlight the relationships observed in exotic and translocated fish species, respectively.

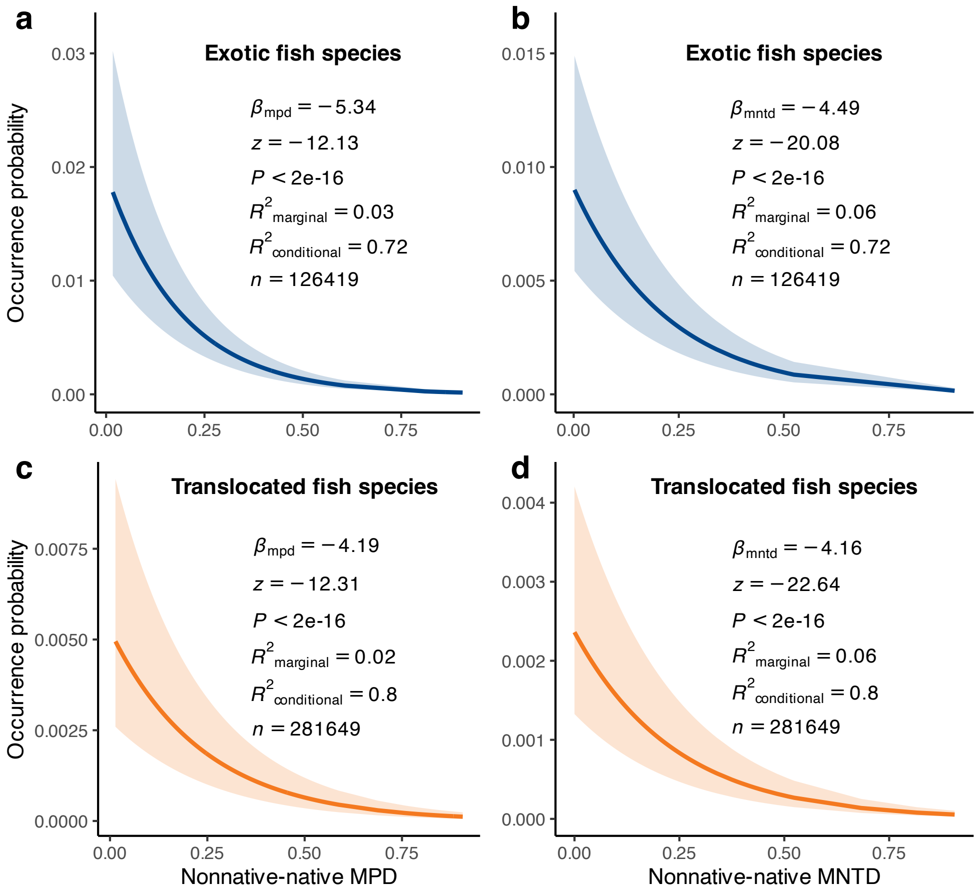

Fig. S4 | Relationships between the probability of non-native fish occurrence and nonnative-native phylogenetic distance after redefining the exotic and translocated non-native species on the biogeographical realm scale. a The variation in the occurrence probability of exotic fish species with the MPD between exotic and native fish species. **b** The variation in the occurrence probability of exotic fish species with the MNTD between exotic and native fish species. **c** The variation in the occurrence probability of translocated fish species with the MPD between translocated and native fish species. **d** The variation in the occurrence probability of translocated fish species with the MNTD between translocated and native fish species. Statistical tests and predictive curves (with 95% confidence intervals) were obtained using generalized linear mixed models (GLMMs), while assuming a binomial error distribution. Statistical significance (*P* values), variance explained ($R^2_{marginal}$ for the fixed effect and $R^2_{conditional}$ for both the fixed and random effects), and sample size (*n*) are presented in the figure. Blue and orange colors are used to highlight the relationships observed in exotic and translocated fish species, respectively.

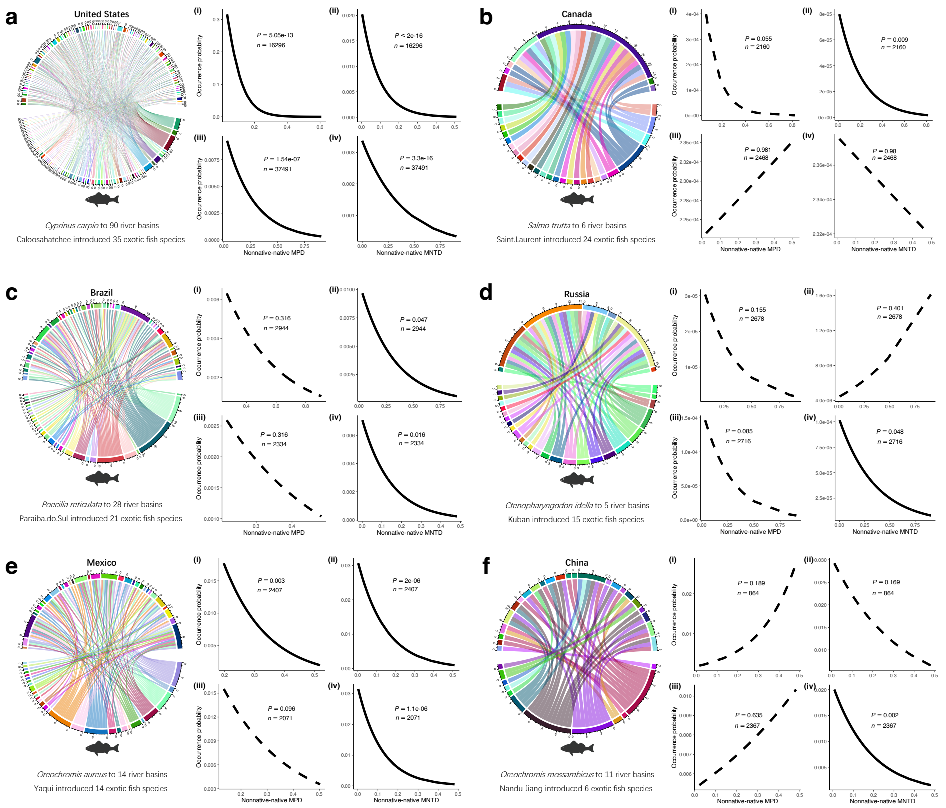

Fig. S5 | Relationships between the probability of non-native fish occurrence and nonnative-native phylogenetic distance in six countries with the largest number of non-native fishes (a United States, b Canada, c Brazil, d Russia, e Mexico, and f China). For each country, **i** the variation in the occurrence probability of exotic fish species with the MPD between exotic and native fish species, **ii** the variation in the occurrence probability of exotic fish species with the MNTD between exotic and native fish species, **iii** the variation in the occurrence probability of translocated fish species with the MPD between translocated and native fish species, and **iv** the variation in the occurrence probability of translocated fish species with the MNTD between translocated and native fish species. Statistical tests and predictive curves were obtained using generalized linear mixed models (GLMMs) while assuming a binomial error distribution, with solid lines representing significant effects ($P < 0.05$). Statistical significance (P values) and sample size (n) are presented in the figure. For each country, a chord diagram illustrates the network relationship between river basins and exotic fish species, showing which exotic fishes have been introduced into specific river basins and which river basins have introduced specific exotic fish species. The most frequently introduced exotic fish species and the river basin that introduced the largest number of exotic fish species are noted below the chord diagrams for clarity.

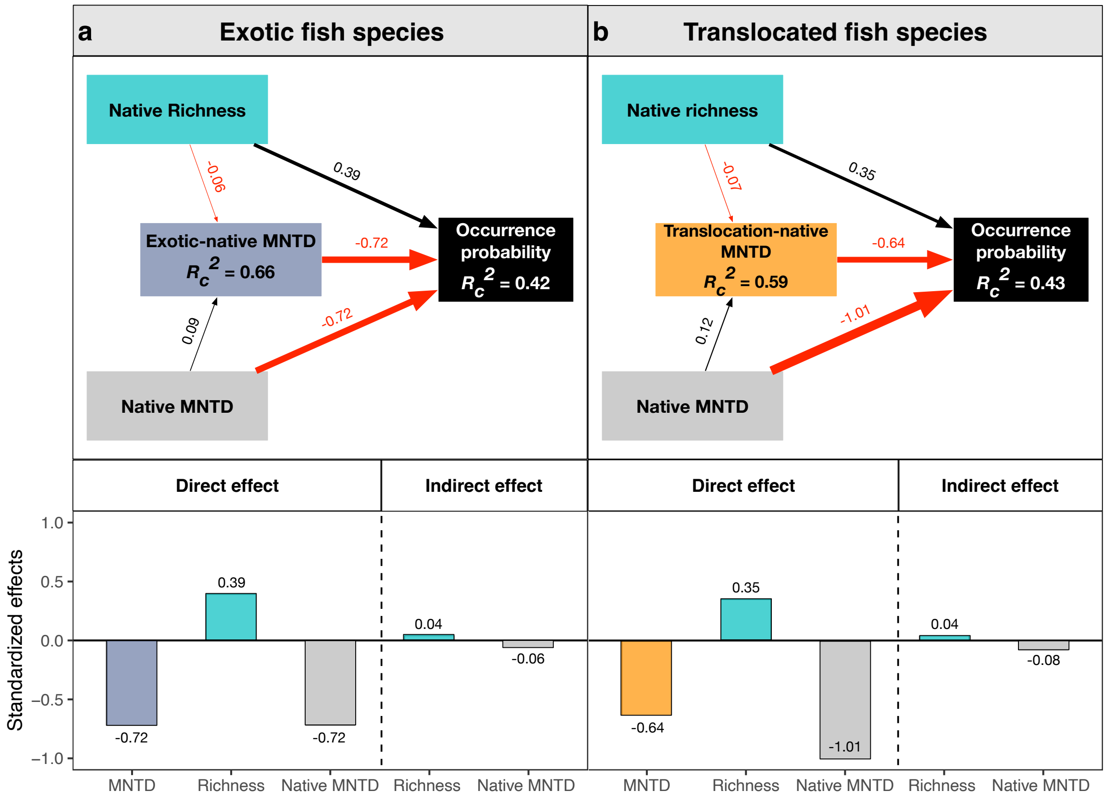

Fig. S6 | Bayesian structural equation modeling for assessing direct and indirect effects of nonnative-native MNTD, native species richness, and native phylogenetic diversity (native MNTD) on occurrence probability of exotic fish species (a) and translocated fish species (b). Boxes represent measured variables, highlighted with distinct colors, while arrows represent relationships among variables. Black and red arrows denote positive and negative effects, respectively. Dashed and solid lines denote 95% credible intervals overlapping with zero or not, respectively. Standardized path coefficients are provided for each significant path, with the width of the path scaled to reflect the magnitude of the standardized path coefficient. The Bayesian conditional R_c^2 (based upon both fixed and random effects) for the endogenous variable is reported in the corresponding boxes. The direct and indirect effects are calculated and presented in the lower part of each panel.

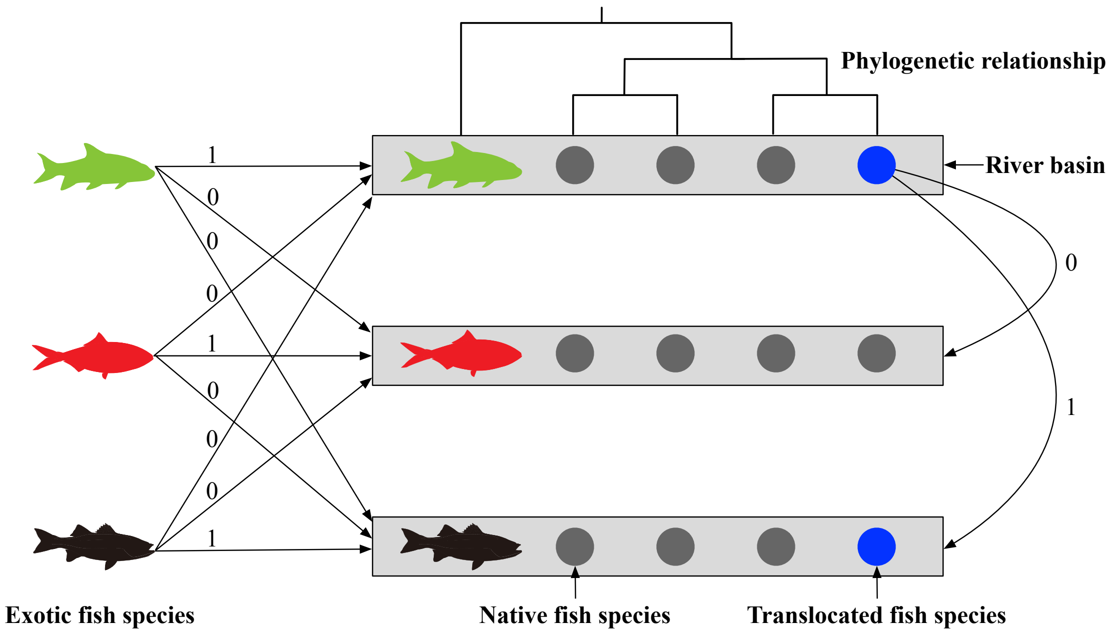

Fig. S7 | Conceptual diagram illustrating the approach to associate nonnative-native phylogenetic distances with non-native fish occurrence. For each exotic fish species, its presence in one (or multiple) river basin within a country is defined as a success (1), while its absence in all the other basins within that country is considered as a failure (0). Similarly, for each translocated fish species (*i.e.* recorded as native species in other river basins within a country), its presence in one (or multiple) river basin is denoted as a success (1), and its absence in all the other basins within the country is considered as a failure (0). For each non-native fish species in a country, its phylogenetic distance with all native species in each river basin within the country is calculated, regardless of whether the non-native species occurs in that basin. This approach establishes the corresponding connection between nonnative-native phylogenetic distance and the conditions of non-native fish occurrence, enabling the examination of the relationship between phylogenetic relatedness and the likelihood of non-native fish occurrence.

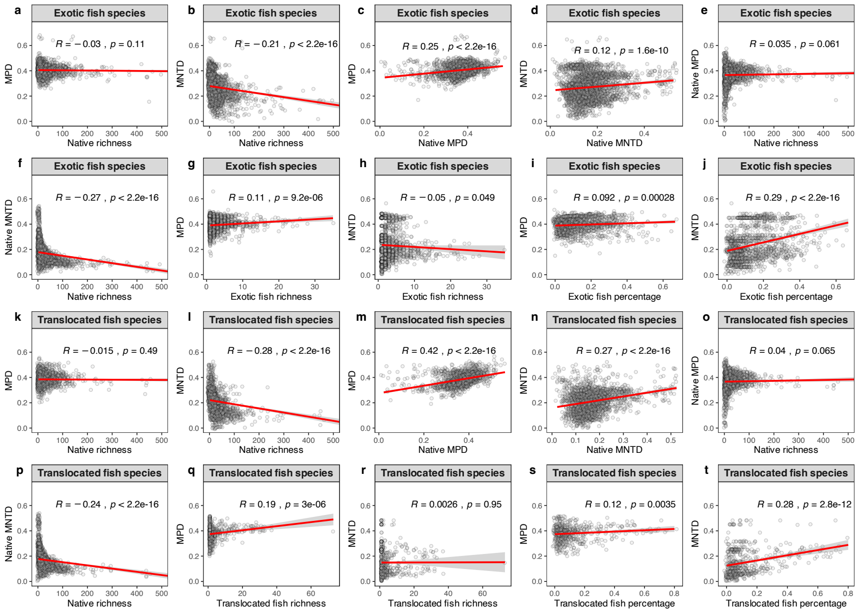

Fig. S8 | Assessment of correlation relationships among key predictor variables. For exotic fish species: **a** the relationship between native richness and MPD; **b** the relationship between native richness and MNTD; **c** the relationship between native MPD and MPD; **d** the relationship between native MNTD and MNTD; **e** the relationship between native richness and native MPD; **f** the relationship between native richness and native MNTD; **g** the relationship between exotic richness and MPD; **h** the relationship between exotic richness and MNTD; **i** the relationship between exotic percentage and MPD; and **j** the relationship between exotic percentage and MNTD. For translocated fish species: **k** the relationship between native richness and MPD; **l** the relationship between native richness and MNTD; **m** the relationship between native MPD and MPD; **n** the relationship between native MNTD and MNTD; **o** the relationship between native richness and native MPD; **p** the relationship between native richness and native MNTD; **q** the relationship between translocated richness and MPD; **r** the relationship between translocated richness and MNTD; **s** the relationship between translocated percentage and MPD; and **t** the relationship between translocated percentage and MNTD. Statistical tests and predictive curves (with 95% confidence intervals) were obtained using general linear models. Correlation coefficient (*R*) and statistical significance (*P* value) are presented in the figure.

Table S1 | Parameter estimates for models explaining the occurrence of exotic and translocated fish species in 3008 river basins worldwide. In these models, nonnative-native mean phylogenetic distance (MPD) and nearest phylogenetic distance (MNTD) were included as separate fixed predictors, with species and river basin nested within the country treated as random effects. An additional phylogenetic covariance matrix was incorporated into these models to account for the phylogenetic non-independence of samples. The statistical tests were conducted using Bayesian phylogenetic mixed models with default priors, while assuming a binomial error distribution. Significant effects (based on whether 95% credible intervals overlap with zero) are highlighted in bold.

Type	Model	Predictor	Mean	SD	0.025 quantile	0.975 quantile
Exotic fishes	1	**Exotic-native MPD**	**-6.78**	**0.42**	**-7.60**	**-5.96**
	2	**Exotic-native MNTD**	**-5.37**	**0.23**	**-5.82**	**-4.91**
Translocated fishes	1	**Translocation-native MPD**	**-4.96**	**0.67**	**-6.27**	**-3.64**
	2	**Translocation-native MNTD**	**-4.50**	**0.31**	**-5.11**	**-3.90**

Table S2 | Marginal effects of phylogenetic relatedness in models explaining the occurrence of exotic and translocated fish species in 3008 river basins worldwide. In these models, nonnative-native MPD and MNTD were included as separate fixed predictors, with basin area serving as the covariate. Species and river basin nested within the country were treated as random effects. The statistical tests were conducted using generalized linear mixed models (GLMMs), while assuming a binomial error distribution. Significant effects ($P < 0.05$) are highlighted in bold.

Type	Model	Predictor	Estimate	*z*	*P*
Exotic fishes	1	**Exotic-native MPD**	**-5.11**	**-15.36**	**< 2e-16**
	2	**Exotic-native MNTD**	**-4.07**	**-22.05**	**< 2e-16**
Translocated fishes	1	**Translocation-native MPD**	**-3.73**	**-7.12**	**1.1e-12**
	2	**Translocation-native MNTD**	**-3.25**	**-12.59**	**< 2e-16**

Table S3 | Marginal effects of diversity in models explaining the occurrence of exotic and translocated fish species in 3008 river basins worldwide. In these models, native species richness, native MPD, and native MNTD were included as separate fixed predictors, with basin area serving as the covariate. Species and river basin nested within the country were treated as random effects. The statistical tests were conducted using generalized linear mixed models (GLMMs), while assuming a binomial error distribution. Significant effects ($P < 0.05$) are highlighted in bold.

Type	Model	Predictor	Estimate	*z*	*P*
Exotic fishes	1	**Native species richness**	**0.003**	**6.48**	**9.35e-11**
	2	**Native MPD**	**2.98**	**4.61**	**4.03e-06**
	3	**Native MNTD**	**-5.32**	**-9.47**	**<2e-16**
Translocated fishes	1	**Native species richness**	**0.003**	**3.66**	**2.52e-04**
	2	**Native MPD**	**10.52**	**7.60**	**2.88e-14**
	3	**Native MNTD**	**-4.99**	**-4.15**	**3.33e-05**
