# Supplementary material for: Global freshwater fish invasion linked to the presence of closely related species
Source: Nat Commun. 2024 Feb 15;15:1411. doi: 10.1038/s41467-024-45736-8 (PMC10869807; doi:10.1038/s41467-024-45736-8)
Supplement: Supplementary file 3 — Reporting Summary [file 41467_2024_45736_MOESM3_ESM.pdf]

nature portfolio

Corresponding author(s): Meng Xu

Last updated by author(s): Jan 26, 2024

Reporting Summary

Nature Portfolio wishes to improve the reproducibility of the work that we publish. This form provides structure for consistency and transparency in reporting. For further information on Nature Portfolio policies, see our Editorial Policies and the Editorial Policy Checklist.

Statistics

For all statistical analyses, confirm that the following items are present in the figure legend, table legend, main text, or Methods section.

n/a	Confirmed	
☐	☒	The exact sample size (n) for each experimental group/condition, given as a discrete number and unit of measurement
☐	☒	A statement on whether measurements were taken from distinct samples or whether the same sample was measured repeatedly
☐	☒	The statistical test(s) used AND whether they are one- or two-sided<br>*Only common tests should be described solely by name; describe more complex techniques in the Methods section.*
☐	☒	A description of all covariates tested
☐	☒	A description of any assumptions or corrections, such as tests of normality and adjustment for multiple comparisons
☐	☒	A full description of the statistical parameters including central tendency (e.g. means) or other basic estimates (e.g. regression coefficient) AND variation (e.g. standard deviation) or associated estimates of uncertainty (e.g. confidence intervals)
☐	☒	For null hypothesis testing, the test statistic (e.g. F, t, r) with confidence intervals, effect sizes, degrees of freedom and P value noted<br>*Give P values as exact values whenever suitable.*
☐	☒	For Bayesian analysis, information on the choice of priors and Markov chain Monte Carlo settings
☒	☐	For hierarchical and complex designs, identification of the appropriate level for tests and full reporting of outcomes
☒	☐	Estimates of effect sizes (e.g. Cohen's d, Pearson's r), indicating how they were calculated

Our web collection on statistics for biologists contains articles on many of the points above.

Software and code

Policy information about availability of computer code

Data collection	No software was used.
Data analysis	R language (4.3.0 version). The entire R codes that support the findings of this study are available at Figshare https://doi.org/10.6084/m9.figshare.25058651

For manuscripts utilizing custom algorithms or software that are central to the research but not yet described in published literature, software must be made available to editors and reviewers. We strongly encourage code deposition in a community repository (e.g. GitHub). See the Nature Portfolio guidelines for submitting code & software for further information.

Data

Policy information about availability of data

All manuscripts must include a data availability statement. This statement should provide the following information, where applicable:

- Accession codes, unique identifiers, or web links for publicly available datasets
- A description of any restrictions on data availability
- For clinical datasets or third party data, please ensure that the statement adheres to our policy

The data that support the findings of this study are available at https://doi.org/10.6084/m9.figshare.25058651. Original freshwater fish occurrence database is available at https://doi.org/10.6084/m9.figshare.c.3739145.v1.

Research involving human participants, their data, or biological material

Policy information about studies with human participants or human data. See also policy information about sex, gender (identity/presentation), and sexual orientation and race, ethnicity and racism.

Reporting on sex and gender	not applicable
Reporting on race, ethnicity, or other socially relevant groupings	not applicable
Population characteristics	not applicable
Recruitment	not applicable
Ethics oversight	not applicable

Note that full information on the approval of the study protocol must also be provided in the manuscript.

Field-specific reporting

Please select the one below that is the best fit for your research. If you are not sure, read the appropriate sections before making your selection.

☐ Life sciences ☐ Behavioural & social sciences ☒ Ecological, evolutionary & environmental sciences

For a reference copy of the document with all sections, see nature.com/documents/nr-reporting-summary-flat.pdf

Ecological, evolutionary & environmental sciences study design

All studies must disclose on these points even when the disclosure is negative.

Study description	We used the most comprehensive freshwater fish occurrence database to explore the biogeographical patterns of non-native fishes and their phylogenetic correlates across the world. We initially presented global biogeographical patterns for both exotic and translocated fish species. Subsequently, we constructed a global phylogenetic tree of freshwater fish species to quantify the phylogenetic distances between each exotic or translocated fish species and native fishes in each river basin within a country. Thereby, we examined the relationship between nonnative-native phylogenetic relatedness and the probability of non-native fish occurrence at global, biogeographical realm, and country scales, respectively. We also examined whether the relationship between phylogenetic relatedness and the occurrence of non-native fish species remains robust when considering the influence of native diversity. We found that non-native species phylogenetically close to native species were more likely to establish in freshwater fish community worldwide.
Research sample	The original freshwater fish occurrence database documents the occurrence of 14953 fish species across 3119 river basins in 143 countries, covering more than 80% of the Earth's continental surface. The database has been published in the journal Scientific Data and can be available at https://doi.org/10.6084/m9.figshare.c.3739145.v1. Based on the occurrence database, we constructed a phylogenetic tree for all fish species in the database, quantified the phylogenetic distance between non-native and native fish species, and analyzed the relationship between occurrence probability of non-native species and phylogenetic relatedness.
Sampling strategy	An extensive survey of literature was conducted since 2003. This survey was complemented with web-based sources from national and international biodiversity inventory initiatives compiling either or both collection and field sampling data.
Data collection	Data collection was done by the author Pablo A. Tedesco et al. The database has been published in 2017 in the journal Scientific Data and can be available at https://doi.org/10.6084/m9.figshare.c.3739145.v1.
Timing and spatial scale	The data was collected from 1960 to 2014 across the globe.
Data exclusions	We excluded 22 countries where no non-native species occurred and those species that were not included in the constructed phylogenetic tree in order to characterize the phylogenetic distance between non-native and native fish species.
Reproducibility	Data and codes for the analysis are available to reproduce all the results.
Randomization	Determining the native or non-native status of species based on their records in the fish occurrence database. Translocated non-native fish species were identified by checking whether a non-native species in a river basin of a country has also been recorded as native species in other basins within the country. Exotic fish species were determined by excluding translocated species from the non-native species lists of each river basin.
Blinding	Blinding was not relevant.

Did the study involve field work? ☐ Yes ☒ No

Reporting for specific materials, systems and methods

We require information from authors about some types of materials, experimental systems and methods used in many studies. Here, indicate whether each material, system or method listed is relevant to your study. If you are not sure if a list item applies to your research, read the appropriate section before selecting a response.

Materials & experimental systems

n/a	Involved in the study
☒	☐ Antibodies
☒	☐ Eukaryotic cell lines
☒	☐ Palaeontology and archaeology
☒	☐ Animals and other organisms
☒	☐ Clinical data
☒	☐ Dual use research of concern
☒	☐ Plants

Methods

n/a	Involved in the study
☒	☐ ChIP-seq
☒	☐ Flow cytometry
☒	☐ MRI-based neuroimaging
